# *CompositIA*: an open-source automated quantification tool for body composition scores from thoraco-abdominal CT scans

**DOI:** 10.1186/s41747-025-00552-7

**Published:** 2025-01-29

**Authors:** Raffaella Fiamma Cabini, Andrea Cozzi, Svenja Leu, Benedikt Thelen, Rolf Krause, Filippo Del Grande, Diego Ulisse Pizzagalli, Stefania Maria Rita Rizzo

**Affiliations:** 1 Euler Institute, Università della Svizzera italiana, Lugano, Switzerland; 2 International Center of Advanced Computing in Medicine (ICAM), Pavia, Italy; 3 Imaging Institute of Southern Switzerland, Ente Ospedaliero Cantonale, Lugano, Switzerland; 4 Faculty of Biomedical Sciences, Università della Svizzera italiana, Lugano, Switzerland

**Keywords:** Body composition, Computed tomography, Deep learning, Open-source software, Segmentation

## Abstract

**Background:**

Body composition scores allow for quantifying the volume and physical properties of specific tissues. However, their manual calculation is time-consuming and prone to human error. This study aims to develop and validate *CompositIA*, an automated, open-source pipeline for quantifying body composition scores from thoraco-abdominal computed tomography (CT) scans.

**Methods:**

A retrospective dataset of 205 contrast-enhanced thoraco-abdominal CT examinations was used for training, while 54 scans from a publicly available dataset were used for independent testing. Two radiology residents performed manual segmentation, identifying the centers of the L1 and L3 vertebrae and segmenting the corresponding axial slices. MultiResUNet was used to identify CT slices intersecting the L1 and L3 vertebrae, and its performance was evaluated using the mean absolute error (MAE). Two U-nets were used to segment the axial slices, with performance evaluated through the volumetric Dice similarity coefficient (vDSC). *CompositIA*’s performance in quantifying body composition indices was assessed using mean percentage relative error (PRE), regression, and Bland–Altman analyses.

**Results:**

On the independent dataset, *CompositIA* achieved a MAE of about 5 mm in detecting slices intersecting the L1 and L3 vertebrae, with a MAE < 10 mm in at least 85% of cases and a vDSC greater than 0.85 in segmenting axial slices. Regression and Bland–Altman analyses demonstrated a strong linear relationship and good agreement between automated and manual scores (*p* values < 0.001 for all indices), with mean PREs ranging from 5.13% to 15.18%.

**Conclusion:**

*CompositIA* facilitated the automated quantification of body composition scores, achieving high precision in independent testing.

**Relevance statement:**

*CompositIA* is an automated, open-source pipeline for quantifying body composition indices from CT scans, simplifying clinical assessments, and expanding their applicability.

**Key Points:**

- Manual body composition assessment from CTs is time-consuming and prone to errors.
- *CompositIA* was trained on 205 CT scans and tested on 54 scans.
- *CompositIA* demonstrated mean percentage relative errors under 15% compared to manual indices.
- *CompositIA* simplifies body composition assessment through an artificial intelligence-driven and open-source pipeline.

**Graphical Abstract:**

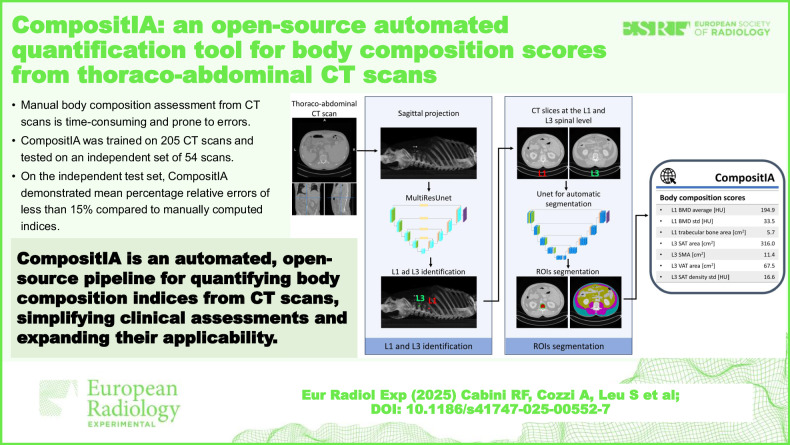

## Background

The assessment of body composition is being progressively integrated into many clinical settings due to its prognostic value for several adverse clinical outcomes [1–3], typically including quantification of the distribution and density of fat, muscle, and bone [4]. This can be noninvasively obtained with different imaging modalities such as computed tomography (CT), which has become almost ubiquitarian in routine clinical practice [5, 6]. As body composition-related information contained in CT scans goes frequently unused—being unrelated to the clinical indication of the CT examination—there is a growing interest in the possibility of using CT examinations as an opportunistic screening modality for a personalized evaluation of the risk of future adverse clinical outcomes [4, 7].

State-of-the-art software allows to perform body composition analysis from CT scans [7, 8], specifically by measuring visceral adipose tissue (VAT), subcutaneous adipose tissue (SAT), and skeletal muscle area (SMA) at the level of the third lumbar vertebra (L3), and bone mineral density (BMD) at the level of the first lumbar vertebra (L1). However, most of these tools rely on manual (or, at best, semiautomated) delineation, making their use time-consuming and preventing the inclusion of body composition analysis in a routine clinical workflow.

Conversely, tools for automated CT-based body composition analysis—able to select the appropriate vertebral levels and perform automatic segmentations—might offer a feasible option to implement the assessment of body composition in routine clinical practice. Recent studies have demonstrated that artificial intelligence methods based on deep learning (DL) can achieve excellent results in the segmentation of chest and abdominal CT images [7, 9–11]. However, at least two general limitations hinder the widespread adoption of DL-based automated body composition analysis. First, open-source DL-based body composition systems are not readily available, and the most popular options currently are proprietary. Additionally, ensuring the portability of DL software across various hardware configurations poses significant challenges.

Thus, this study aimed to: (i) develop an automatic method able to select the appropriate axial images on L1 and L3 for subsequent body composition segmentations; (ii) integrate a second method that automatically segments the regions of interest for body composition assessment, specifically on L1 for BMD and at the L3 level for SAT, VAT, and SMA. As a secondary objective, we aimed to release the open-source software for research purposes.

## Methods

### Training set

This retrospective single-center study was conducted at the Imaging Institute of Southern Switzerland (Ente Ospedaliero Cantonale, Lugano, Switzerland) after specific approval from the local ethics committee (Comitato Etico Cantonale, Repubblica e Cantone Ticino, Switzerland; protocol code 2021-00943). To build the training set, we retrieved contrast-enhanced thoraco-abdominal CT examinations of inpatients and outpatients, performed between January 2019 and December 2021 on one of the six CT scanners of our institution (one Somatom Definition Flash and four Somatom Definition Edge, Siemens Healthineers, Erlangen, Germany; one Brilliance ICT, Philips Healthcare, Eindhoven, the Netherlands). The CT scans were acquired according to a reference of image quality, and the tube voltage kV was automatically adapted by the CT scanners, all equipped with automatic tube current modulation systems. For the purposes of this study, we considered for inclusion only the venous phase acquired with a 3-mm slice thickness, excluding CT scans with evident acquisition problems (noise or motion artifacts) and those with the presence of spinal metallic implants near the L1 and L3 vertebrae. From the 242 thoraco-abdominal CT scans acquired with a venous phase and a slice thickness of 3 mm, 37 were excluded due to the presence of motion artifacts or spinal metallic implants, ultimately leading to the inclusion of 205 examinations from 205 patients (97 females and 108 males; median age 70.4 years, interquartile range 57.0–78.3 years). The pixel size was 0.76 mm ± 0.09 mm (mean ± standard deviation).

### Independent test set

To evaluate the performance of *CompositIA* on an external dataset entirely independent from the training data, we used a public dataset [12] providing CT scans from routine clinical studies [13]. We selected 54 thoraco-abdominal CT scans having a field of view comparable with the scans included in the training set—according to the aforementioned inclusion criteria—but acquired with different scanners, slice thicknesses, resolutions, contrast phases, kVp, and reconstruction kernels.

### Manual segmentation

The annotation process was performed by a radiology resident with 4 years of experience in CT (for the training set) and by another radiology resident with 5 years of experience in CT (for the independent test set) using the ITK-SNAP segmentation tool [14]. Both residents consulted a senior radiologist (with 20 years of experience in CT interpretation) in cases of uncertainty. Initially, the L1 and L3 vertebrae were identified, and the corresponding CT slices at the level of the transverse processes of the two vertebrae were extracted. Subsequently, the regions of interest were delineated as follows:

- At the level of the L1 vertebra, the trabecular bone and cortical regions were segmented in accordance with previous evidence about the significant correlation of CT-derived trabecular bone density at this level with T-scores at dual-energy x-ray absorptiometry [15]. Although the cortical region does not contribute to the quantification of any body composition index, it was segmented to ensure it would not subsequently be included by the DL model in the trabecular bone region;
- At the level of the L3 vertebra, the VAT, SAT, and SMA regions were segmented, again according to established evidence about the most reliable site to assess these volumes [16–18].

### *CompositIA* pipeline

**Fig. 1:**
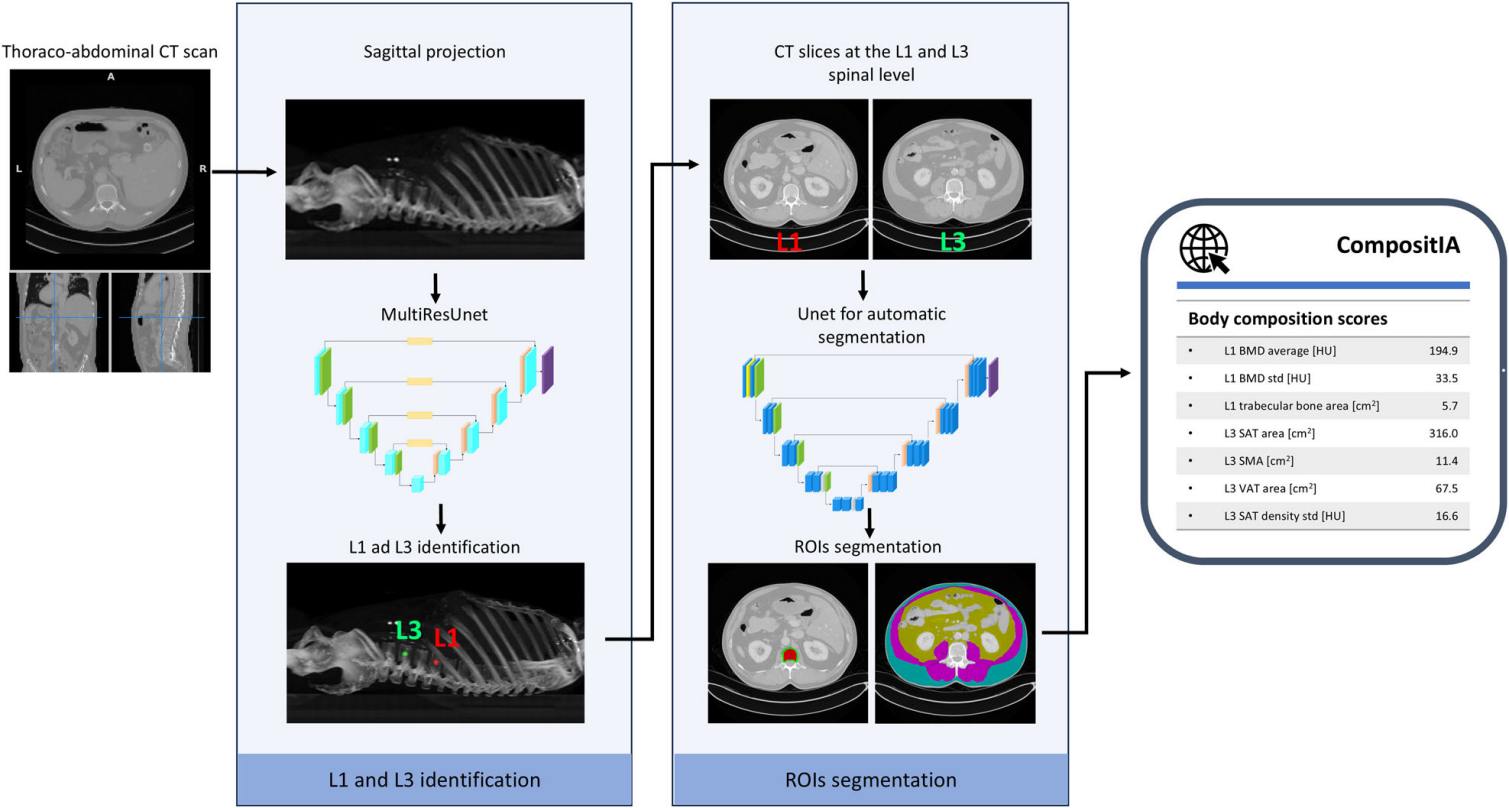
Overview of the *CompositIA* system for calculating body composition indices from thoraco-abdominal CT scans. The pipeline includes automatic identification of L1 and L3 vertebrae, segmentation of image slices at the L1 and L3 spinal level, and quantification of body composition indices. The system employs MultiResUNet and U-net DL models for identification and segmentation tasks, respectively. BMD, Bone mineral density; CT, Computed tomography; L1, First lumbar vertebra; L3, Third lumbar vertebra; SAT, Subcutaneous adipose tissue; SMA, Skeletal muscle area; VAT, Visceral adipose tissue, ROI, Region of interest

The analysis pipeline, hereafter referred to as *CompositIA* system, automatically computes body composition indices from thoraco-abdominal CT scans. It consists of three components: (i) automatic identification of L1 and L3 vertebrae; (ii) segmentation of image slices at the L1 and L3 level; and (iii) quantification of body composition indices. A summary of our image analysis pipeline is depicted in Fig. 1.

#### L1 and L3 identification

The problem of localizing the centers of the L1 and L3 vertebrae was approached as an image generation task. As a preliminary step, the 3D CT images were projected onto their 2D sagittal projections and resampled into isotropic voxels. The projections were then windowed using three HU ranges of [1,000, 2,000], [400, 500], and [800, 1,900], forming three-channel images. These images were then normalized to the range [0, 1] and further reshaped into 128 × 256 pixel arrays. The objective of the vertebral localization problem is to create an image where the centers of L1 and L3 are highlighted by clusters of increasing intensity, with values set to 1 at their respective centers, while all other pixels are set to 0. This image was generated by applying a Gaussian filter with a σ of 15 to an image obtained by setting the pixels identifying the centers of the L1 and L3 vertebrae to 1 and the remaining pixels to 0. To tackle this task, the MultiResUNet architecture was used [19], as described in Fig. S1 of the Supplementary Material. To finally determine the positions of the L1 and L3 vertebral centers, we analyzed the predicted images and attributed the positions of the two most intense peaks to the centers of L1 and L3.

#### L1 and L3 slices segmentation

To segment the CT slices at both the L1 and L3 vertebral levels, we used a DL method based on U-net [20]. Specifically, two U-nets (U-net_L1_ and U-net_L3_) were designed for segmenting the trabecular bone and cortical regions of the L1 vertebra and the SMA, VAT, and SAT regions at the level of L3. A detailed description of the models can be found in Fig. S2 of the Supplementary Material. Both U-nets were trained using CT slices composed of 512 × 512 pixels. However, different grayscale preprocessing steps were applied to the L1 and L3 level images to enhance the relevant structures for their respective segmentation tasks. Specifically, the L1 level slice was windowed using the HU range of [-1,024, 500] to enhance the contrast of the bone. For the L3 level slice, three different windowing settings were applied with HU ranges of [-1,024, 2,048], [-190, -30], and [40, 100]. The first range preserved the original image contrast, the second range highlighted adipose tissue, while the third emphasized muscular tissue. The resulting images were then combined into a three-channel image, which was used as input for the U-net_L3_. Images from both U-nets were normalized through linear scaling to fit within the [0, 1] range.

### Definition of body composition indices

The *CompositIA* system produces the following body composition indices as output:

- BMD average (HU): the mean density of the trabecular bone region in HU at the L1 vertebral level;
- BMD standard deviation (HU): the standard deviation of the density of the trabecular bone region in HU at the L1 vertebral level;
- L1 trabecular bone area (cm²): the total area of the trabecular bone region in cm² at the L1 vertebral level;
- L3 SAT area (cm²): the total area of the SAT region in cm² at the L3 vertebral level;
- L3 SMA (cm²): the total area of the SMA region in cm² at the L3 vertebral level;
- L3 VAT area (cm²): the total area of the VAT region in cm² at the L3 vertebral level;
- L3 SAT density standard deviation (HU): the standard deviation of the density of the SAT region in HU at the L3 vertebral level.

### Training and evaluation strategies for the DL models

All DL models that constitute the *CompositIA* system were trained, validated, and tested by using a *k*-fold cross-validation with *k* = 5, iteratively using a different fold as the test set and the remaining *k* − 1 folds as the training set. The overall model performance on the internal dataset was assessed by calculating the mean performance across the five hold-out folds. This strategy was used to evaluate both the performance of individual DL models and the overall effectiveness of the *CompositIA* system.

The overall performance of the *CompositIA* system on the independent dataset was tested after retraining all the models on the complete training dataset, combining the five folds together.

The different steps of *CompositIA* were evaluated as follows:

1. The performance of L1 and L3 center detection was assessed using mean absolute error (MAE) to measure the average error in millimeters and assessing the percentage of cases where the error was less than 10 millimeters;
2. Segmentation performance was evaluated using the volumetric Dice similarity coefficient (vDSC) to measure the overlap between the predicted and ground truth segmentations (vDSC = 1, perfect overlap; vDSC = 0, the two masks are completely disjoint);
3. The accuracy of body composition indices estimation was assessed using mean percentage relative error, linear regression analyses, and Bland–Altman analysis.

## Results

### L1 and L3 detection performance

**Fig. 2:**
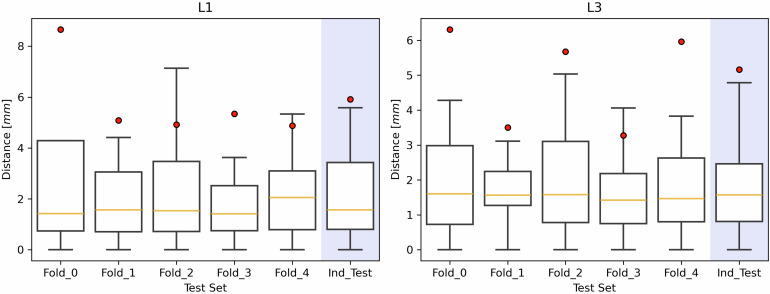
L1 and L3 detection performance: absolute distance (mm) between the ground truth and the predicted centers of L1 and L3 vertebrae for each fold and for the independent test set (Ind_Test), highlighted in blue. Red dots represent the mean values of the distributions. L1, First lumbar vertebra; L3, Third lumbar vertebra

The MultiResUNet’s performance in identifying L1 and L3 was first evaluated using the MAE across the five hold-out folds (Fig. 2). For L1 center localization, the MAE was 5.8 ± 1.6 mm, and the mean slice error was 2 ± 1. For L3 center localization, the MAE was 4.9 ± 1.4 mm, and the mean slice error was 2 ± 1. The errors were under 10 mm in 88% ± 1% of cases for L1 and 89% ± 1% of cases for L3.

**Fig. 3:**
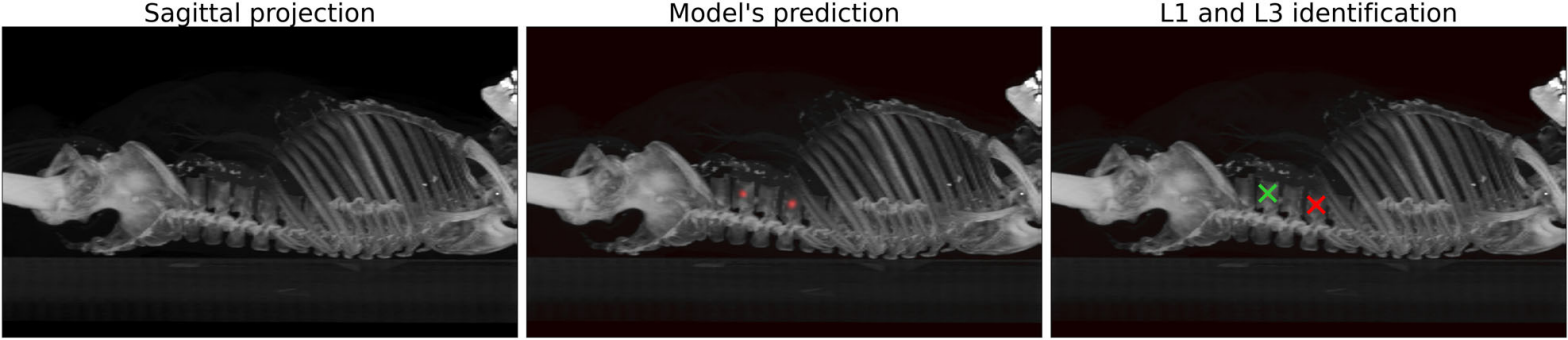
Results of the L1 and L3 vertebral center identification model. In the left panel, the input sagittal projection is represented. In the center panel, the output of the MultiResUNet is overlaid on the input projection. On the right panel, the L1 and L3 centers are determined from the network’s output. L1, First lumbar vertebra; L3, Third lumbar vertebra

On the independent test set (Fig. 2), the MAE for L1 center localization was 5.9 ± 10.2 mm with a mean slice error of 4 ± 7, while for L3 the MAE was 5.2 ± 10.3 mm with a mean slice error of 4 ± 7. The percentage of cases where the error was less than 10 mm was 85% for L1, and it was 89% for L3. Figure 3 illustrates the DL model prediction, with additional examples provided in Fig. S5 of the Supplementary Material.

### L1 and L3 segmentation performance

**Fig. 4:**
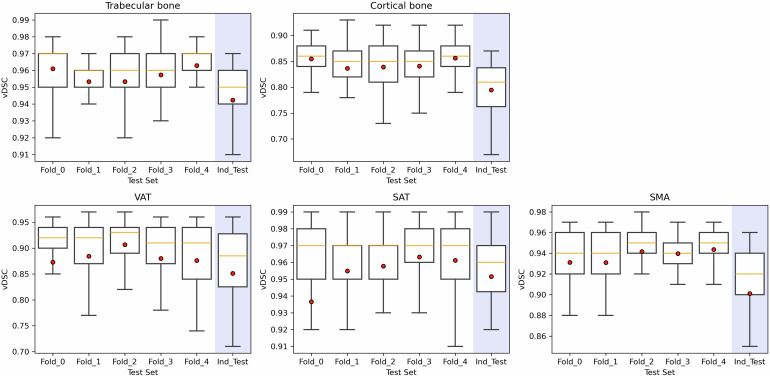
U-net_L1_ and U-net_L3_ segmentation performance. The first row shows the vDSC (volumetric Dice Similarity Coefficient) for trabecular bone and cortical segmentations for each test fold and for the independent test set (Ind_Test), highlighted in blue. The second row shows the vDSC for VAT, SAT, and SMA segmentations for each test fold. Red dots represent the mean values of the vDSC distributions. SAT, Subcutaneous adipose tissue; SMA, Skeletal muscle area; VAT, Visceral adipose tissue; vDSC, Volumetric Dice similarity coefficient

The segmentation performance of the two models (Fig. 4) was first assessed using the vDSC averaged on the 5 hold-out folds. U-net_L1_ achieved a vDSC of 0.96 ± 0.01 for the trabecular bone region and a vDSC of 0.85 ± 0.01 for the cortical region. U-net_L3_ achieved a vDSC of 0.94 ± 0.01 for SMA, a vDSC of 0.89 ± 0.01 for VAT, and a vDSC of 0.96 ± 0.01 for SAT.

**Fig. 5:**
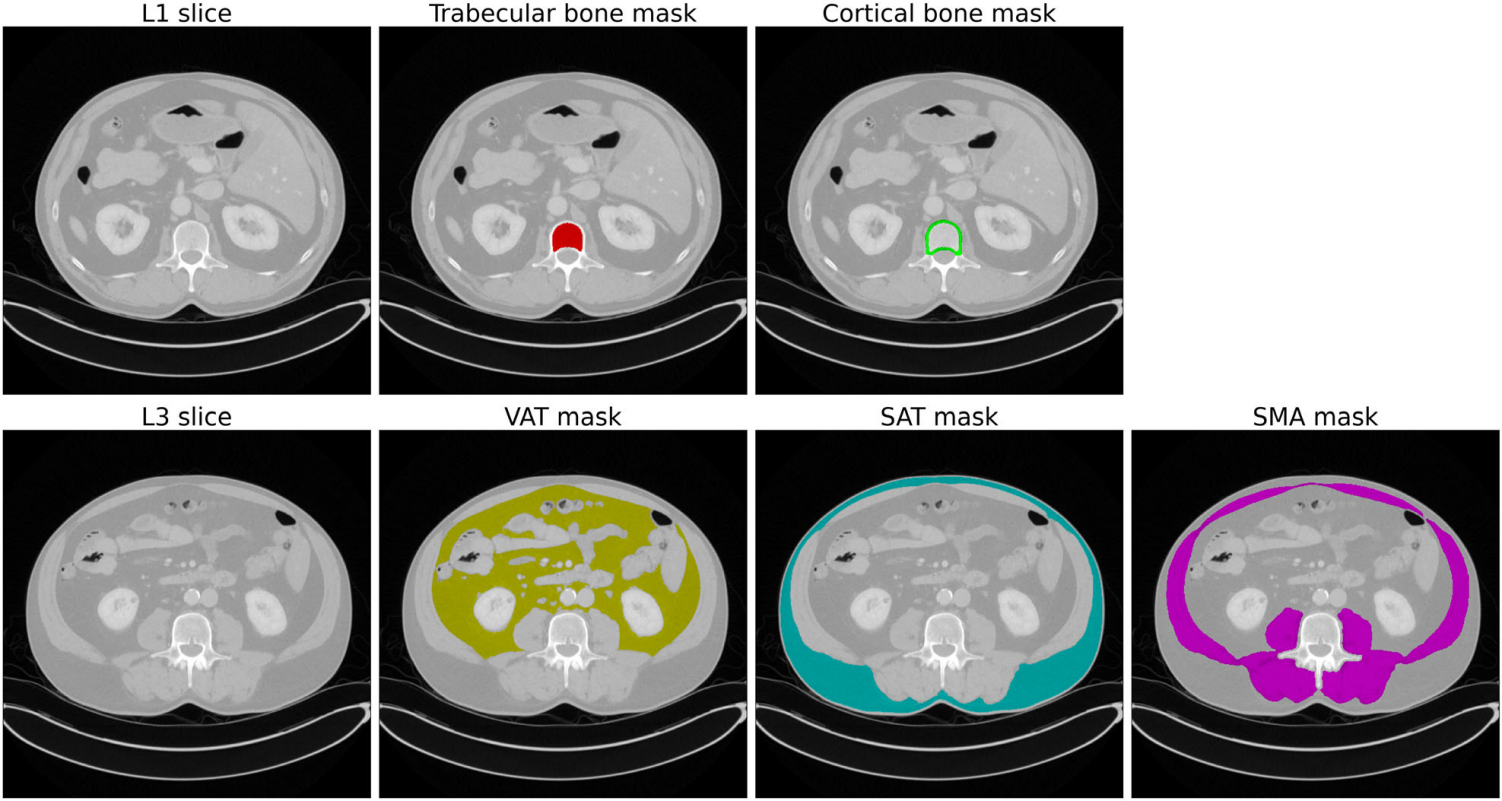
*CompositIA* segmentation masks: the top row displays the U-net_L1_ output tailored for the segmentation of the trabecular bone and cortical regions of the L1 vertebra, while the bottom row represents the U-net_L3_ output, with a focus on segmenting the SMA, VAT, and SAT regions. L1, First lumbar vertebra; L3, Third lumbar vertebra; SAT, Subcutaneous adipose tissue; SMA, Skeletal muscle area; VAT, Visceral adipose tissue

On the independent dataset (as shown in Fig. 4), U-net_L1_ obtained a mean vDSC of 0.94 ± 0.02 for trabecular bone segmentation and 0.79 ± 0.06 for cortical segmentation. U-net_L3_ achieved a mean vDSC of 0.90 ± 0.06 for SMA, 0.85 ± 0.11 for VAT, and 0.95 ± 0.03 for SAT. Figure 5 illustrates U-net_L1_ and U-net_L3_ predictions, with additional examples provided in Fig. S6 of the Supplementary Material.

### *CompositIA* performance in quantifying body composition indices

**Fig. 6:**
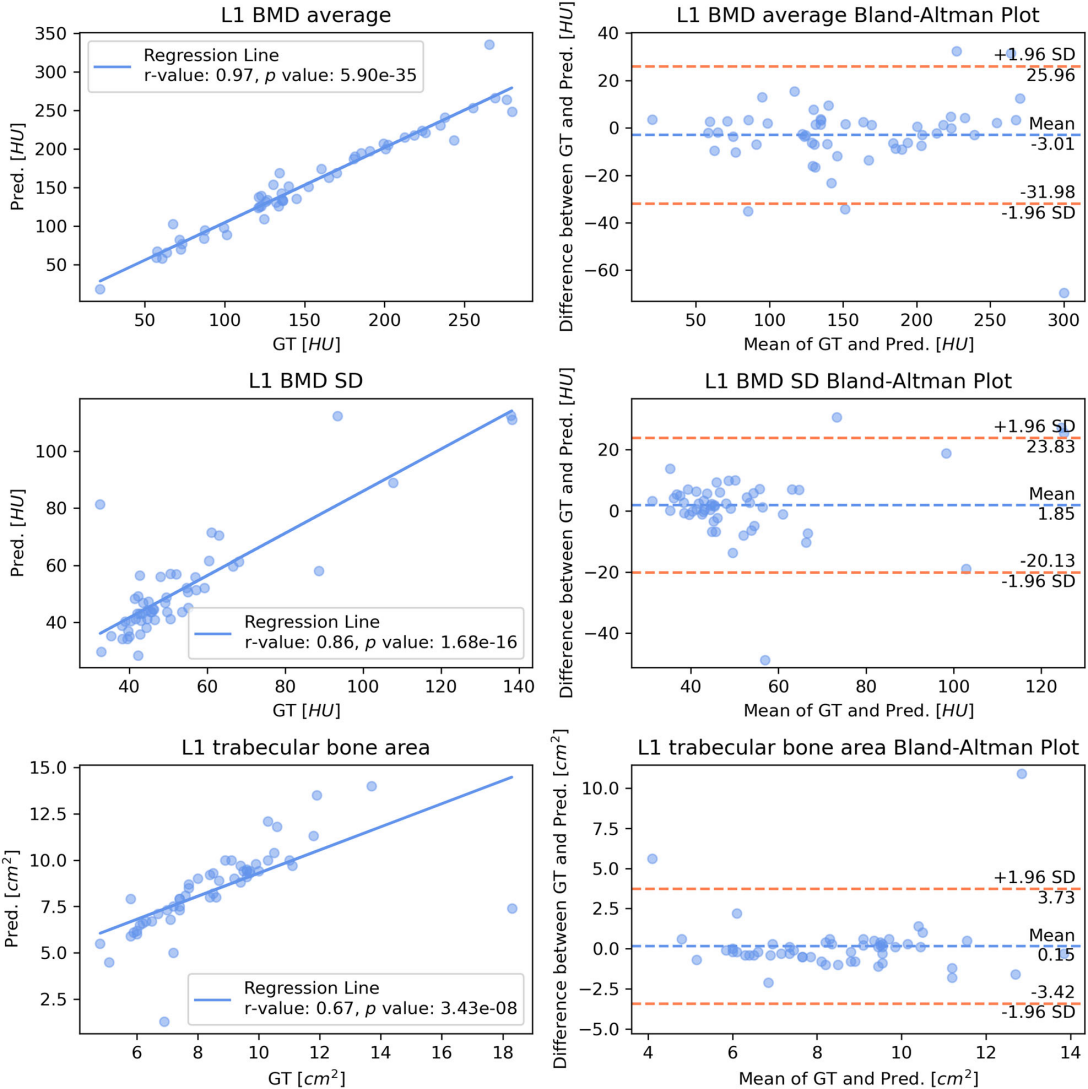
Comparison of *CompositIA*-predicted (Pred) values and ground truth (GT) for the three L1 trabecular bone indexes on the independent test set: L1 trabecular bone density average (HU), L1 trabecular bone density standard deviation (HU), and L1 trabecular bone area (cm²). The left panels show scatter plots of GT *versus* Pred, including the regression line, the Pearson correlation coefficient (*r*-value), the associated *p* value, and the coefficient of determination (*r*² value). The right panels show Bland–Altman plots, which visually represent the agreement between GT and Pred. BMD, Bone mineral density; L1, First lumbar vertebra; SD, Standard deviation

**Fig. 7:**
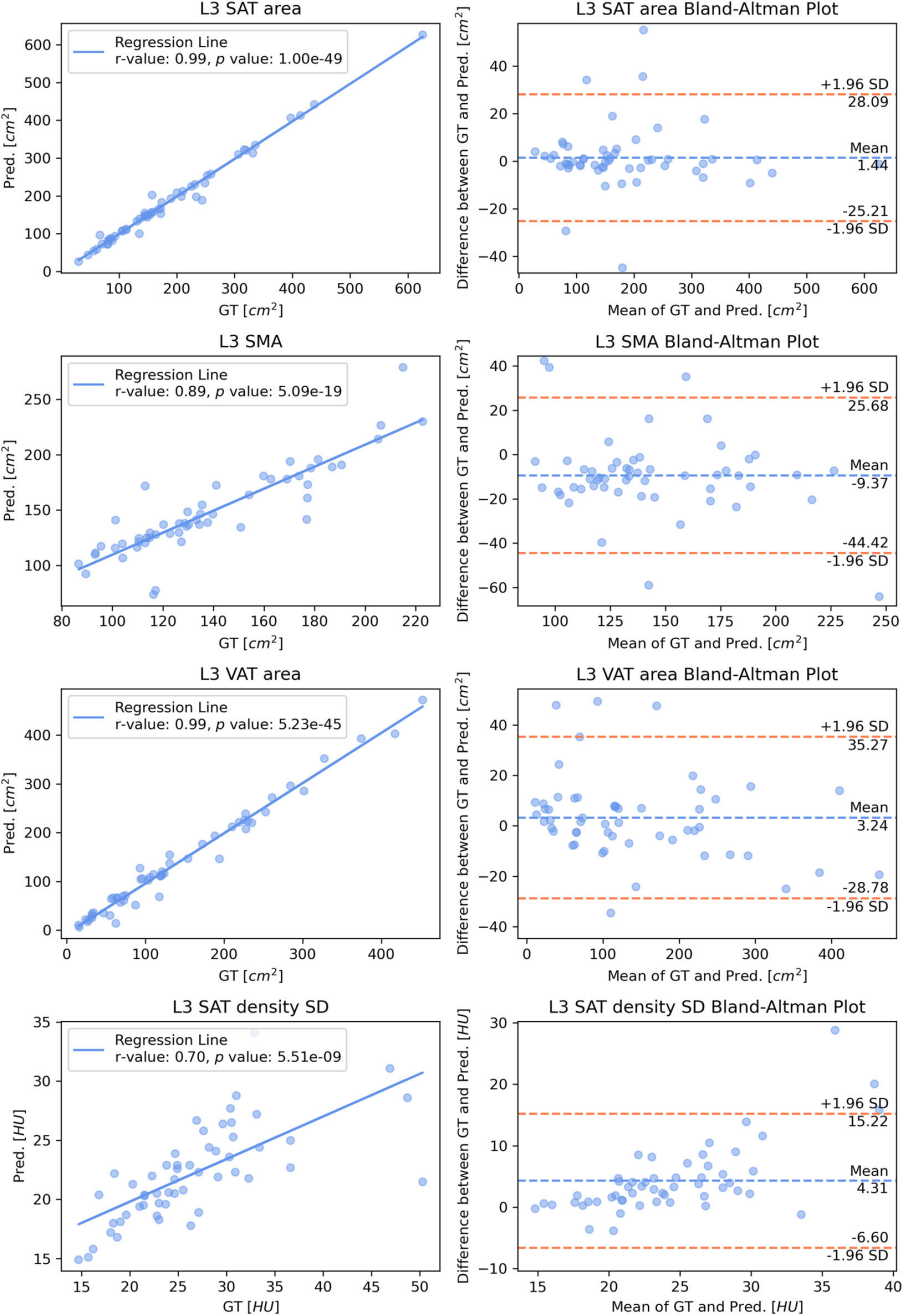
Comparison of *CompositIA*-predicted (Pred) values and ground truth (GT) and for the four L3 Indexes on the independent test set: L3 SAT area (cm²), L3 SMA (cm²), L3 VAT area (cm²) and L3 SAT density standard deviation (HU). The left panels show scatter plots of GT *versus* Pred, including the regression line, the Pearson correlation coefficient (*r*-value), the associated *p* value, and the coefficient of determination (*r*² value). The right panels show Bland–Altman plots, which visually represent the agreement between Pred and GT. L3, Third lumbar vertebra; SAT, Subcutaneous adipose tissue; SD, Standard deviation; SMA, Skeletal muscle area; VAT, Visceral adipose tissue

Table 1 summarizes the results of linear regression and Bland–Altman analyses for the aggregated 5 hold-out folds. When considering the independent test set, a strong positive linear relationship was observed for all the body composition indices examined in this study (Figs. 6 and 7). Pearson correlation coefficients (*r*-values) ranged from 0.86 to 0.99, with slight deviations noted for the density standard deviations of the L1 trabecular bone and of the L3 SAT, where the coefficients were 0.67 and 0.70, respectively. All Pearson correlation coefficients were statistically significant with *p* values < 0.001. High *r*² values ranging from 0.73 to 0.99 were observed for the L1 trabecular bone density average, L1 trabecular bone area, L3 SAT area, L3 SMA, and L3 VAT area. Additionally, a moderate positive correlation was noted between the L1 trabecular bone density standard deviation (*r*² = 0.45) and the L3 SAT density standard deviation (*r*² = 0.48).

**Table 1:** Comparison of *CompositIA*-predicted values and ground truth and for the L1 and L3 indexes in the *k*-fold cross-validation

	*k*-fold cross-validation						
Body composition indexes	*r*	*p* value	*r*²	Bias	Upper LOA	Lower LOA	PRE ± SD (%)
L1 trabecular BMD average (HU)	0.98	5.90e-35	0.95	-1.49	21.33	-24.30	4.35 ± 7.60
L1 trabecular BMD standard deviation (HU)	0.76	1.68e-16	0.57	0.23	18.08	-17.63	10.51 ± 14.71
L1 trabecular bone area (cm²)	0.89	3.43e-08	0.79	0.08	1.88	-1.71	6.16 ± 8.81
L3 SAT area (cm²)	0.99	8.73e-161	0.97	0.05	33.75	-33.64	6.38 ± 8.78
L3 SMA (cm²)	0.96	1.82e-109	0.91	1.32	23.39	-20.74	5.25 ± 6.10
L3 VAT area (cm²)	0.99	6.89e-183	0.98	-4.86	25.93	-35.65	10.35 ± 13.43
L3 SAT density standard deviation (HU)	0.64	3.17e-25	0.41	2.16	10.82	-6.50	11.60 ± 9.57

*BMD* Bone mineral density, *L1* First lumbar vertebra, *L3* Third lumbar vertebra, *LOA* Limit of agreement, *SAT* Subcutaneous adipose tissue, *SMA* Skeletal muscle area, *VAT* Visceral adipose tissue, *PRE* Percentage relative error, *SD* Standard deviation

Bland–Altman analyses (Figs. 6 and 7) demonstrated good agreement between the *CompositIA* system’s predicted values and ground truth values for body composition indices. Specifically, the average differences were near zero, ranging from -3 to 4 HU for density indexes and from 0.15 to 3.24 cm² for area indexes, except for the SMA, which had an average difference of -9.37 cm². The limits of agreement ranges were narrow for all indices.

The mean percentage relative errors (Table 2) were below 15% for L1 trabecular bone density average (6.98%), L1 trabecular bone density standard deviation (13.13%), L1 trabecular bone area (9.45%), L3 SAT area (5.13%), L3 SMA (11.73%), and L3 VAT area (13.02%). The mean percentage relative error was slightly higher for the L3 SAT density standard deviation (15.18%).

**Table 2:** Comparison of *CompositIA*-predicted values and ground truth for the L1 and L3 indexes in the independent test set

Body composition indexes	Independent test set						
	*r*	*p *value	*r*²	Bias	Upper LOA	Lower LOA	PRE ± SD (%)
L1 trabecular BMD average (HU)	0.97	5.90e-35	0.95	-3.01	25.96	-31.98	6.98 ± 8.77
L1 trabecular BMD standard deviation (HU)	0.86	1.68e-16	0.73	1.85	23.83	-20.13	13.13 ± 20.69
L1 trabecular bone area (cm²)	0.67	3.43e-08	0.45	0.15	3.73	-3.42	9.45 ± 13.93
L3 SAT area (cm²)	0.99	1.00e-49	0.99	1.44	28.09	-25.21	5.13 ± 8.20
L3 SMA (cm²)	0.89	5.09e-19	0.79	-9.37	25.68	-44.42	11.73 ± 10.45
L3 VAT area (cm²)	0.99	5.23e-45	0.98	3.24	35.27	-28.78	13.02 ± 15.89
L3 SAT density standard deviation (HU)	0.70	5.51e-09	0.48	4.31	15.22	-6.60	15.18 ± 11.94

*BMD* Bone mineral density, *L1* First lumbar vertebra, *L3* Third lumbar vertebra, *LOA* Limit of agreement, *SAT* Subcutaneous adipose tissue, *SMA* Skeletal muscle area, *VAT* Visceral adipose tissue, *PRE* Percentage relative error, *SD* Standard deviation

### Comparison with other architectures

Considering the importance of accurate identification of L1 and L3 centers on body composition estimation, we systematically compared our method with a set of different DL architectures across five hold-out folds. As detailed in Figs. S3 and S4 of the Supplementary Material, the assessed methods included L1 and L3 centers regression with a voting scheme, MultiResUNet employing Gaussian σ of 5 and 15 (*CompositIA*), MultiResUNet applied to both sagittal and coronal projections and U-net using Gaussian σ of 15. Table 3 provides an overview of the comparisons, highlighting MAE and center classification accuracy.

**Table 3:** Comparison with some other methodologies for the identification of L1 and L3 vertebrae centers

Model	*k*-fold cross-validation			
	MAE L1 (mm)	MAE L3 (mm)	Perc. MAE < 10 mm L1 (%)	Perc. MAE < 10 mm L3 (%)
Regression and voting scheme	10.7 ± 1.8	10.6 ± 1.8	58% ± 11%	64% ± 1%
U-net *σ* = 15	Loss does not decrease			
MultiResUNet *σ* = 5	25.7 ± 14.0	15.3 ± 6.0	55% ± 7%	72% ± 6%
MultiResUNet sagittal and coronal	6.4 ± 1.8	6.8 ± 2.5	83% ± 1%	84% ± 1%
MultiResUNet *σ* = 15 (*CompositIA*)	5.8 ± 1.6	4.9 ± 1.4	88% ± 1%	89% ± 1%

Metrics were evaluated through the *k*-fold cross-validation. *MAE* Mean absolute error, *Perc. MAE* < 10 mm percentage of cases with a mean absolute error lower than 10 mm

## Discussion

Our study aimed to develop an automated pipeline to compute body composition scores from thoraco-abdominal CT scans, with three main components: automatic selection of CT slices intersecting the L1 and L3 vertebrae, segmentation of these slices, and quantification of seven different body composition indices.

The system demonstrated a low average localization error (approximately 5 mm) for both the L1 and L3 vertebrae (much smaller than the L1 and L3 average height of 25 mm), with errors under 10 mm in at least 85% of cases. This result outperformed all the considered state-of-the-art methods. Most failures occurred in cases involving patients with prostheses or medical implants, which were underrepresented in the training data and presented CT artifacts.

The system also achieved vDSC values consistently above 0.85 for all the segmentations, except for the cortical region (with a vDSC of approximately 0.79). This lower performance can be attributed to the significant size difference between the small cortical region and the larger background, which poses significant challenges for accurate segmentation of this area compared to others. However, this does not affect *CompositIA*’s overall performance since the cortical region is not used for any body index quantification.

The predicted body composition indices showed strong positive correlations and agreement with ground truth values for most indices, except for L1 trabecular bone density standard deviation and L3 SAT density standard deviation, which were more sensitive to localization errors of the vertebral centers.

We performed a robust cross-validation strategy by testing all *CompositIA* components using both *k*-fold cross-validation and an independent dataset. The minimal performance drop between these evaluations demonstrates the system’s strong generalization capability, despite the differences in acquisition setups and clinical characteristics of the patients in the two datasets.

The fully open-source nature of the *CompositIA* system differentiates it from other body composition analysis software [7, 8], which are typically proprietary and, therefore, less accessible to both clinicians and software developers. *CompositIA* entails three different usage modalities aimed at different user categories: (i) computer vision researchers can access the complete source code to train, test, and modify each step of the pipeline; (ii) clinicians can use *CompositIA* through the web application or (iii) they can download and run the pipeline locally without installing any software on their device.

This work has limitations: first, the training dataset is relatively small, included only venous-phase CT images with a 3 mm slice thickness, and was annotated by a single clinician. While the pipeline was tested on a completely independent dataset containing CT scans acquired with different systems, the generalizability of our findings could be enhanced by testing the model in a multicentric setting, using datasets from different centers while ensuring decentralization [21]. Patients with vertebral fractures, spinal metallic devices, and anatomical variations (*e.g*., scoliosis or six lumbar-type vertebrae) were excluded from both the training and independent test sets, limiting the possibility of assessing *CompositIA*’s performance in these cases. The performance of both the L1 and L3 center identification system and segmentation models showed a slight dependency on patient age, as detailed in Fig. S7 of the Supplementary Material. Increasing the training dataset to include a larger number of older patients could mitigate the impact of age on system performance.

Additionally, although different algorithms for identifying L1 and L3 were evaluated, it would be useful to assess the pipeline’s performance against other state-of-the-art automated body composition tools using the same external dataset. Finally, the segmentation process could be further refined by incorporating interactive modifications through manual region selection.

In conclusion, the results of this study demonstrate that the *CompositIA* system is capable of accurately quantifying body composition scores from thoraco-abdominal CT scans, offering an open-source option to facilitate the access to advanced body composition assessment for both clinical and research applications.

## Supplementary information

**Additional file 1: Fig. S1.** MultiResUNet architecture: the neural network consists of an encoder-decoder structure, each with 4 MutiRes blocks and connected by Residual Paths (Res Path). The encoder (left) has convolutional layers (Conv2D), Rectified Linear Unit (ReLU) activation, and MaxPooling, while the decoder uses convolutional layers, ReLU activation, and Transposed Convolutions. The final layer applies a sigmoid activation for output predictions. In the lower-left corner, the composition of a MultiRes Block is depicted, utilizing consecutive 2D convolutions with 3 × 3 kernels to factorize convolutions of 5 × 5 and 7 × 7 kernels. In the lower-right corner, the composition of the Residual Path with 4 convolutional blocks (ResPath 4). **Fig. S2.** U-net scheme: the neural network consists of an encoder-decoder architecture, each with 4 convolutional blocks. The encoder (left) has convolutional layers, ReLU activation, and MaxPooling, while the decoder uses convolutional layers, ReLU activation, and UpSampling. A middle block connects the encoder and decoder, and the final layer applies softmax for binary segmentations. **Fig. S3.** Overview of the voted regression method employed for the identification of L1 and L3 planes from thoraco-abdominal CT scans. 2D Sagittal projections are computed from 3D volumes. Then, random patches (P) are extracted from each projection (panel a). Each patch is processed through a CNN for regression of the L1 and L3 coordinates (panel b). Values obtained from multiple patches are finally merged via a voting method (panel c). **Fig. S4.** The left panel depicts the input image of the model, which is a combination of sagittal and coronal projections along the *y*-axis. The right panel illustrates the model’s output, composed of the combination of the sagittal and coronal ground truth maps highlighting the L1 and L3 vertebra centers with increasing intensity peaks. **Fig. S5.** Sagittal projections of three representative subjects from the independent test set with L1 and L3 ground truth (GT) and predicted (pred) centers. The left panel shows the subject with the lowest model performance for L1 cortical segmentation (vDSC = 0.53 for the cortical region), the center panel depicts the oldest subject in the dataset, and the right panel highlights a subject with a hip prosthesis. Ground truth and predicted centers for L1 and L3 vertebrae are indicated by distinct colors. **Fig. S6.** Segmentations for three representative subjects from the independent test set. The left panel shows the subject with the lowest model performance for L1 cortical segmentation (vDSC = 0.53 for the cortical region), the center panel depicts the oldest subject in the dataset, and the right panel highlights a subject with a hip prosthesis. The top row displays the ground truth segmentations, while the bottom row shows the segmentations predicted by the models. **Fig. S7.** Detection performance of L1 and L3 vertebrae centers, measured as the absolute distance (mm) between ground truth and predicted centers, as a function of patient age on the independent test set (blue) and on the *k*-fold cross-validation (red). Spearman's correlation coefficient (ρ) and associated *p* values are reported to assess the relationship between age and detection performance. **Fig. S8.** Segmentation performance of U-net_L1_ and U-net_L3_ models, measured by vDSC, as a function of patient age on the independent test set (blue) and on the *k*-fold cross-validation (red). Spearman's correlation coefficient (ρ) and associated *p* values are reported to assess the relationship between age and segmentation accuracy.

## Data Availability

The datasets used and analyzed during the current study are available from the corresponding author upon reasonable request.

## Abbreviations

BMD: Bone mineral density
CT: Computed tomography
DL: Deep learning
L1: First lumbar vertebra
L3: Third lumbar vertebra
MAE: Mean absolute error
SAT: Subcutaneous adipose tissue
SMA: Skeletal muscle area
VAT: Visceral adipose tissue
vDSC: Volumetric Dice similarity coefficient

## Code availability

The modules of the *CompositIA* system have been implemented with Python 3.8, leveraging on Tensorflow 2.8 [22]. The code can be downloaded from: https://github.com/rcabini/compositIA/. We developed a web application with a graphical interface that allows users to upload the CT scan, run the code, and see the results directly from the browser. This has been implemented with HTML/PHP/Javascript and can be executed online at https://www.dp-lab.info/compositia/ or locally through a stand-alone application for Windows. The application provides outputs for all three phases, making it easier to identify system errors and determine their stage. Furthermore, as the step that most influences the body composition accuracy is the localization of L1 and L3, the platform allows users to visualize and manually adjust L1 and L3 positions if necessary. This feature allows users also to apply the pipeline semiautomatically to CT scans with different fields of view than the training dataset. Additionally, users who wish to apply the system to CT datasets acquired using imaging systems distinct from those in the training and independent test sets, can download the code and retrain the models using their own data, calibrating the system to fit their specific dataset.
