# Supplementary material for: CompositIA: an open-source automated quantification tool for body composition scores from thoraco-abdominal CT scans
Source: Eur Radiol Exp. 2025 Jan 29;9:12. doi: 10.1186/s41747-025-00552-7 (PMC11780042; doi:10.1186/s41747-025-00552-7)
Supplement: Supplementary file 1 — Additional file 1: Fig. S1. MultiResUNet architecture: the neural network consists of an encoder-decoder structure, each with 4 MutiRes blocks and connected by Residual Paths (Res Path). The encoder (left) has convolutional layers (Conv2D), Rectified Linear Unit (ReLU) activation, and MaxPooling, while the decoder uses convolutional layers, ReLU activation, and Transposed Convolutions. The final layer applies a sigmoid activation for output predictions. In the lower-left corner, the composition of a MultiRes Block is depicted, utilizing consecutive 2D convolutions with 3 × 3 kernels to factorize convolutions of 5 × 5 and 7 × 7 kernels. In the lower-right corner, the composition of the Residual Path with 4 convolutional blocks (ResPath 4). Fig. S2. U-net scheme: the neural network consists of an encoder-decoder architecture, each with 4 convolutional blocks. The encoder (left) has convolutional layers, ReLU activation, and MaxPooling, while the decoder uses convolutional layers, ReLU activation, and UpSampling. A middle block connects the encoder and decoder, and the final layer applies softmax for binary segmentations. Fig. S3. Overview of the voted regression method employed for the identification of L1 and L3 planes from thoraco-abdominal CT scans. 2D Sagittal projections are computed from 3D volumes. Then, random patches (P) are extracted from each projection (panel a). Each patch is processed through a CNN for regression of the L1 and L3 coordinates (panel b). Values obtained from multiple patches are finally merged via a voting method (panel c). Fig. S4. The left panel depicts the input image of the model, which is a combination of sagittal and coronal projections along the y-axis. The right panel illustrates the model’s output, composed of the combination of the sagittal and coronal ground truth maps highlighting the L1 and L3 vertebra centers with increasing intensity peaks. Fig. S5. Sagittal projections of three representative subjects from the independent t [file 41747_2025_552_MOESM1_ESM.pdf]

CompositIA: an open-source automated quantification tool for body composition scores from thoraco-abdominal CT scans

ELECTRONIC SUPPLEMENTARY MATERIAL

MultiResUNet for L1 and L3 identification

To tackle the L1 and L3 identification task, the MultiResUNet architecture was used [1]. MultiResUNet is a DL architecture that enhances the basic U-Net architecture [2] by utilizing MultiRes blocks that enable the extraction of information at different scale levels. Each MultiRes block comprises successive 3×3 and 1×1 convolutions, Rectified Linear Unit (ReLu) activation, and batch normalization, to factorize convolutions of 5×5 and 7×7 kernels. The model is structured as an encoder-decoder system interconnected by residual blocks. The encoder part includes four MultiRes blocks followed by a 2×2 MaxPooling layer to reduce spatial dimensions, while the decoder comprises four MultiRes blocks followed by 2×2 Transposed Convolution layers to increase spatial dimensions. A single MultiRes block connects the encoder and the decoder. A schematic representation of the model's architecture is represented in Fig. S1.

To train the MultiResUNet, we minimized the mean squared error loss function over 1000 epochs. During training, we monitored the mean absolute error (MAE) and selected the MultiResUNet weights that yielded the lowest MAE. To enhance the diversity of the training data and improve the model's generalization ability, we employed contrast stretching as a data augmentation technique.

UNets for L1 and L3 slices segmentation

U-net$_{L1}$ and U-net$_{L3}$ consisted of an encoder-decoder structure with skip connections that enabled the extraction of both local and global features [2]. The encoder had four down-sampling blocks with two convolutional layers (kernel size 3×3 and ReLU activation function) and a MaxPooling layer (pool size 2×2). The decoder mirrored the encoder's structure, replacing the MaxPooling layer with an UpSampling layer (upsampling factor 2×2). A middle block composed by two convolutional layers connected the encoder and decoder. In the final layer of the U-net, the softmax function was applied to obtain binary segmentations as output. A schematic representation of the architecture is shown in Fig. S2.

The training of the two U-nets was performed for 100 epochs, minimizing the cross-entropy loss function. The volumetric Dice similarity coefficient (vDSC) was used as an additional metric to monitor the training process [3]. We selected the U-net weights that achieved the best vDSC during training. To increase the diversity of the training data and improve the model's ability to generalize

to new samples, we applied data augmentation techniques. These included geometric operations such as rotation, horizontal/vertical flipping, zooming, horizontal/vertical shifting, and shearing.

L1 and L3 centers regression and voting scheme

The problem of localizing the centers of the L1 and L3 vertebrae was addressed as a regression task. As a preliminary step, the 3D CT images were projected onto their 2D sagittal projections and resampled into isotropic voxels. The projections were then windowed using three HU ranges of [1000, 2000], [400, 500], and [800, 1900], forming three-channel images, which were subsequently normalized to the range [0, 1]. The projections were then decomposed into square patches of size 224 mm, each labeled with the distance from its center to the position of the L1 and L3 vertebrae as summarized in Fig. S3(a). The targets of the regression problem are the vectors connecting the centers of the patches to the centers of the L1 and L3 vertebrae.

We used a regression model consisting of two components: the first component is a convolutional module inspired by the architecture of VGG19 [4], the second component consists of a sequence of fully connected layers. Specifically, the VGG19 consists of a total of 16 convolutional layers (kernel size of 3×3 with ReLU activation functions) and 5 MaxPooling layers (pool size of 2×2). The convolutional layers are organized into 5 blocks, each containing a varying number of convolutional layers followed by a MaxPooling layer. The output of VGG19 is then flattened and passed through a sequence of three fully connected layers, with the final layer utilizing a linear activation function for the output predictions. A schematic representation of the model's architecture is represented in Fig. S3(b).

To finally determine the positions of the L1 and L3 vertebral centers, the predicted positions from all image patches are aggregated using a voting scheme. Each patch contributes a +1 vote at the predicted coordinates of the target in the voting space, except for patches that predict regions outside the image which do not contribute to the voting system. By accumulating the votes from all patches, the voting space captures the overall distribution of predicted positions. A gaussian filter is then applied to refine the distribution profile, and the final positions of the L1 and L3 vertebral centers are determined based on the highest vote count. The voting scheme is illustrated in Fig. S3(c).

MultiResUNet applied to sagittal and coronal projections

The coronal and sagittal projections obtained as described in the Methods section were combined along the y-axis, as depicted in Fig. S4. The resultant images were subsequently normalized to the range [0, 1] and reshaped into 256x256 pixel arrays. The ground truth maps depicting the L1 and L3 vertebra centers with increasing intensity peaks were generated by employing the same methodology described in the Methods section for both the coronal and sagittal projection and then combined along the y-axis.

To determine the positions of the L1 and L3 vertebral centers, the predicted image was then split back into two parts along the y-axis. For each of these two images, the positions of the two most

intense peaks were calculated, with the leftmost peak assigned to L1 and the rightmost peak assigned to L3. Finally, the positions obtained from the two projections were averaged.

Results for three representative subjects of the independent test set

We evaluated the performance of the L1 and L3 vertebrae centers localization model (Fig. S5) and the U-netL1 and U-netL3 models (Fig. S6) for three specific non-healthy subjects of the independent test set: the subject with the lowest model performance for L1 cortical segmentation (vDSC = 0.53 for the cortical region), the oldest individual in the dataset, and a subject with a hip prosthesis.

Models' performance as a function of patient age

We evaluated the performance of the localization models for the L1 and L3 vertebrae centers (Fig. S5) and the U-netL1 and U-netL3 models (Fig. S6) as a function of patient age on both the aggregated five hold-out sets and the independent test set. Spearman's correlation coefficient (ρ) and corresponding *p* values were used to assess these correlations. For the aggregated five hold-out sets, significant correlations (p value < 0.05) were observed for the L1 center localization as well as for the segmentations of trabecular, cortical, SAT, and SMA regions. On the independent test set, a significant correlation was found only for SAT segmentation. In all cases where significant correlations were identified, the model errors tended to increase with patient age: specifically, the absolute distance between the predicted and ground true L1 and L3 centers increased as patient age increased, and the vDSC decreased as patient age increased.

References

1. Ibtehaz N, Rahman MS (2020) MultiResUNet: Rethinking the U-Net architecture for multimodal biomedical image segmentation. Neural Netw 121:74–87. https://doi.org/10.1016/j.neunet.2019.08.025
2. Ronneberger O, Fischer P, Brox T (2015) U-Net: convolutional networks for biomedical image segmentation. In: Navab N, Hornegger J, Wells W, Frangi A (eds) Medical image computing and computer-assisted intervention – MICCAI 2015. Lecture Notes in Computer Science(), vol 9351. Springer, Cham, pp 234–241
3. Taha AA, Hanbury A (2015) Metrics for evaluating 3D medical image segmentation: analysis, selection, and tool. BMC Med Imaging 15:29. https://doi.org/10.1186/s12880-015-0068-x
4. Simonyan K, Zisserman A (2014) Very deep convolutional networks for large-scale image recognition. arXiv preprint arXiv:1409.1556. https://doi.org/10.48550/arXiv.1409.1556

Supplementary figures

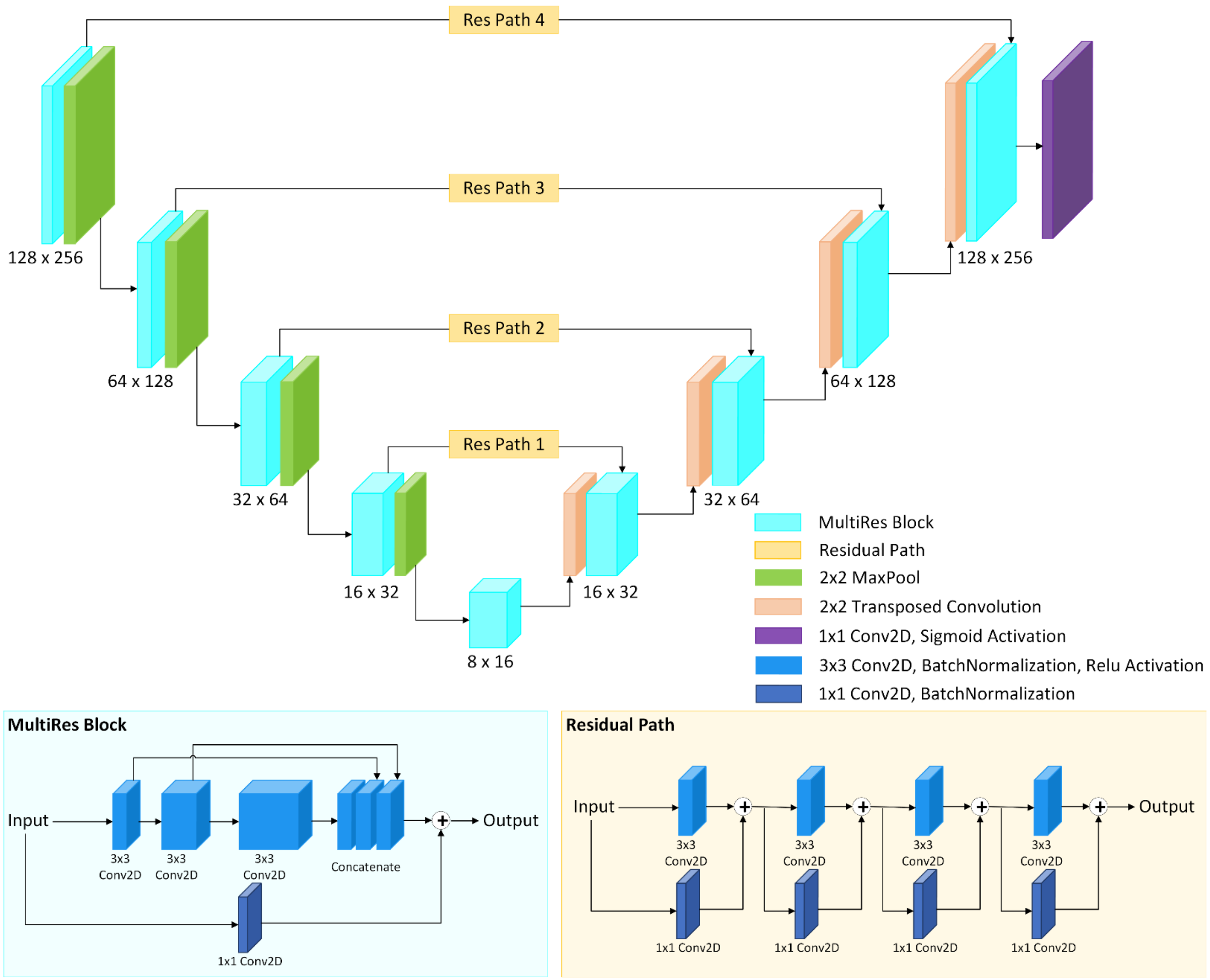

Fig. S1. MultiResUNet architecture: the neural network consists of an encoder-decoder structure, each with 4 MutiRes blocks and connected by Residual Paths (Res Path). The encoder (left) has convolutional layers (Conv2D), Rectified Linear Unit (ReLU) activation, and MaxPooling, while the decoder uses convolutional layers, ReLU activation, and Transposed Convolutions. The final layer applies a sigmoid activation for output predictions. In the lower-left corner, the composition of a MultiRes Block is depicted, utilizing consecutive 2D convolutions with 3x3 kernels to factorize convolutions of 5x5 and 7x7 kernels. On the lower-right corner, the composition of the Residual Path with 4 convolutional blocks (ResPath 4).

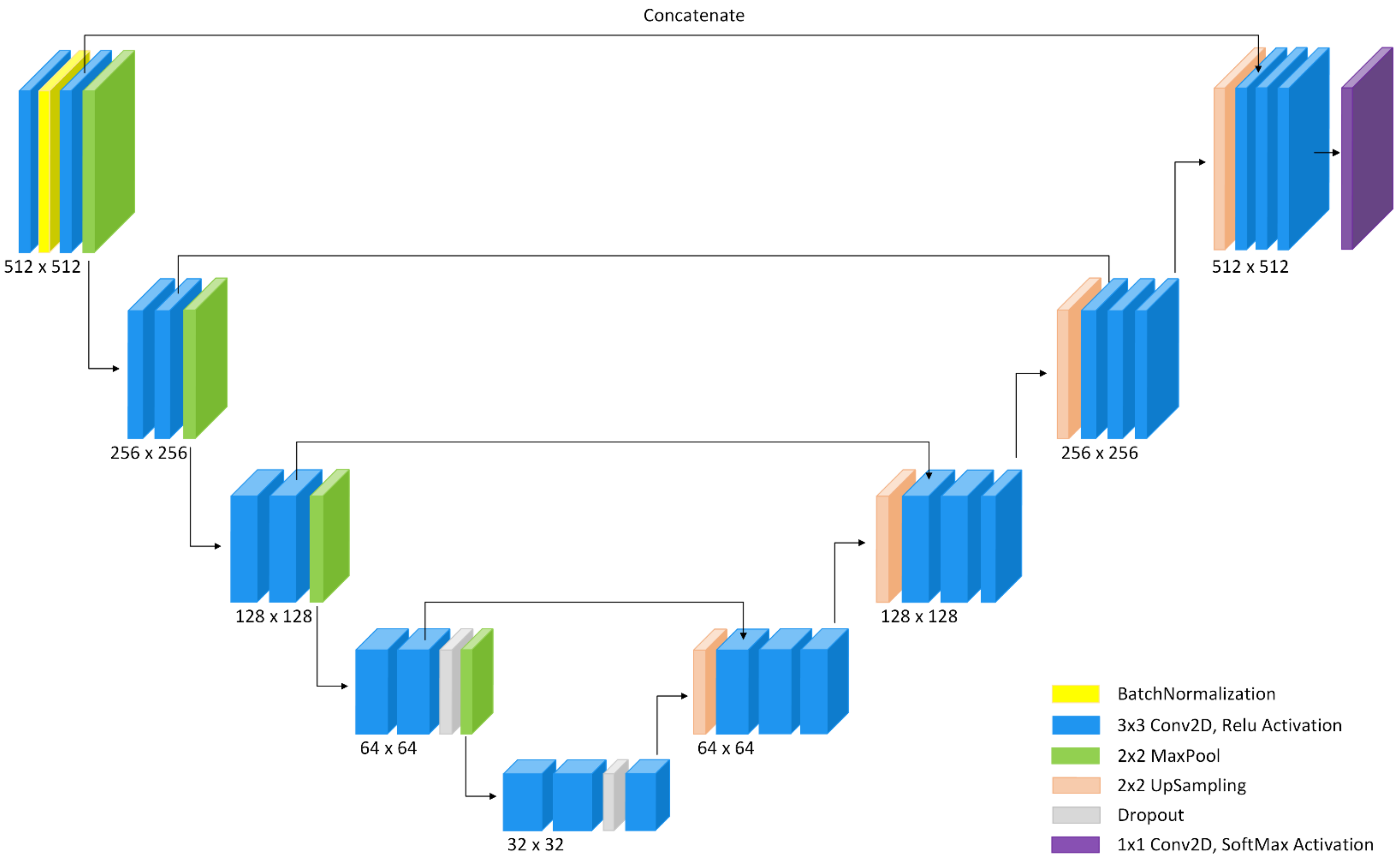

Fig. S2. U-net scheme: the neural network consists of an encoder-decoder architecture, each with 4 convolutional blocks. The encoder (left) has convolutional layers, ReLU activation, and MaxPooling, while the decoder uses convolutional layers, ReLU activation, and UpSampling. A middle block connects the encoder and decoder, and the final layer applies softmax for binary segmentations.

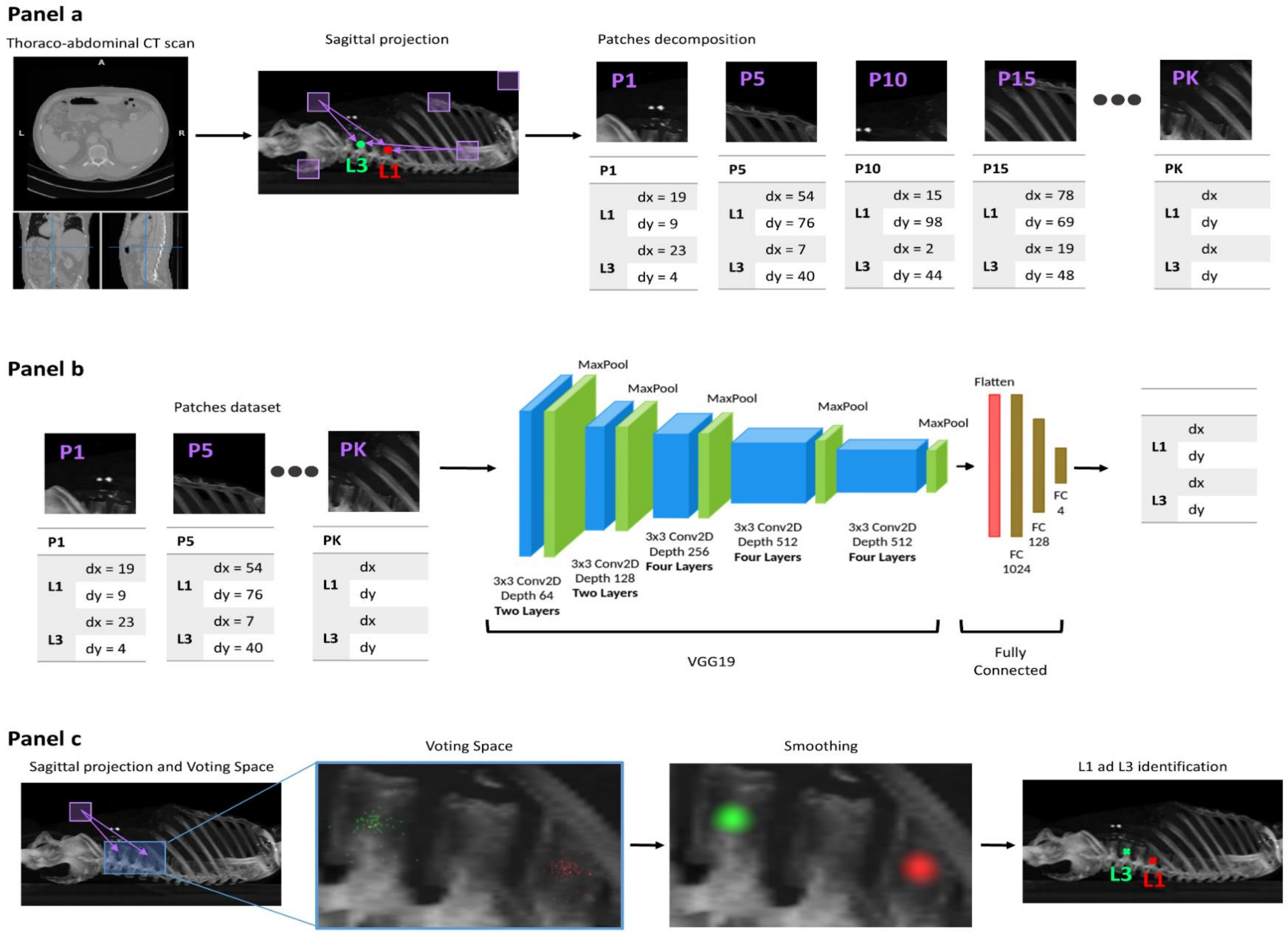

Fig. S3. Overview of the voted regression method employed for the identification of L1 and L3 planes from thoraco-abdominal CT scans. 2D Sagittal projections are computed from 3D volumes. Then, random patches (P) are extracted from each projection (panel a). Each patch is processed through a CNN for regression of the L1 and L3 coordinates (panel b). Values obtained from multiple patches are finally merged via a voting method (panel c).

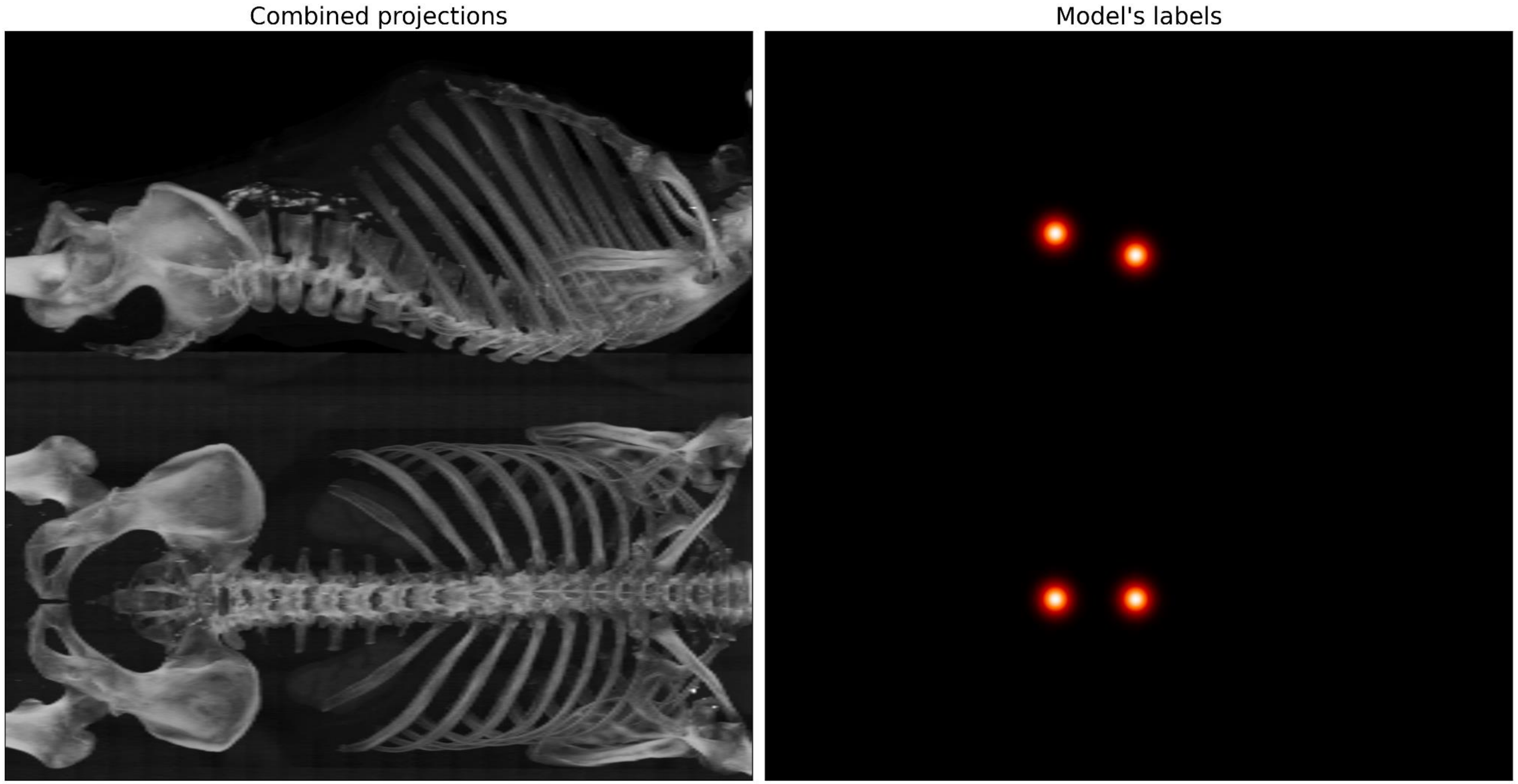

Fig. S4. The left panel depicts the input image of the model, which is a combination of sagittal and coronal projections along the y-axis. The right panel illustrates the model's output, composed by the combination of the sagittal and coronal ground truth maps highlighting the L1 and L3 vertebra centers with increasing intensity peaks.

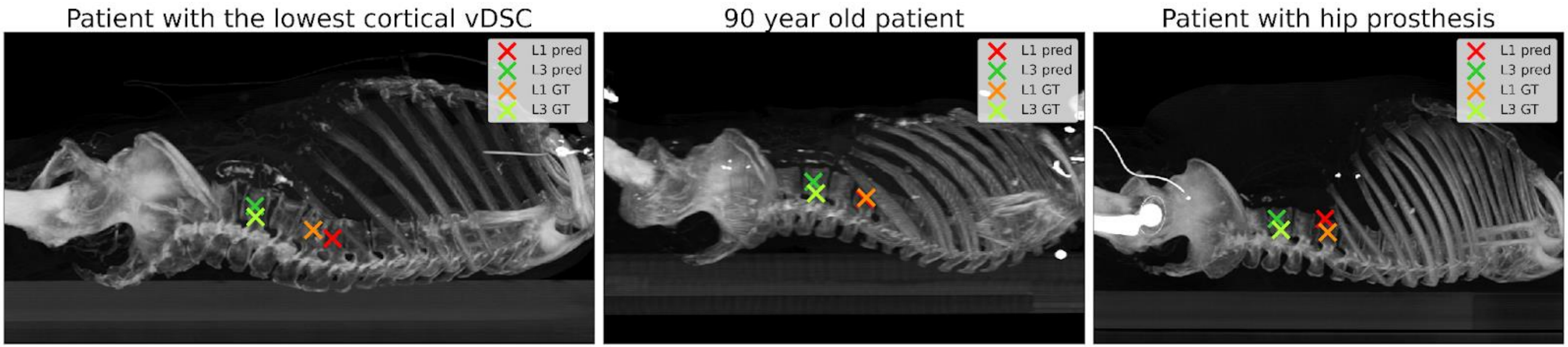

Fig. S5. Sagittal projections of three representative subjects from the independent test set with L1 and L3 ground truth (GT) and predicted (pred) centers. The left panel shows the subject with the lowest model performance for L1 cortical segmentation (vDSC=0.53 for the cortical region), the center panel depicts the oldest subject in the dataset, and the right panel highlights a subject with a hip prosthesis. Ground truth and predicted centers for L1 and L3 vertebrae are indicated by distinct colors.

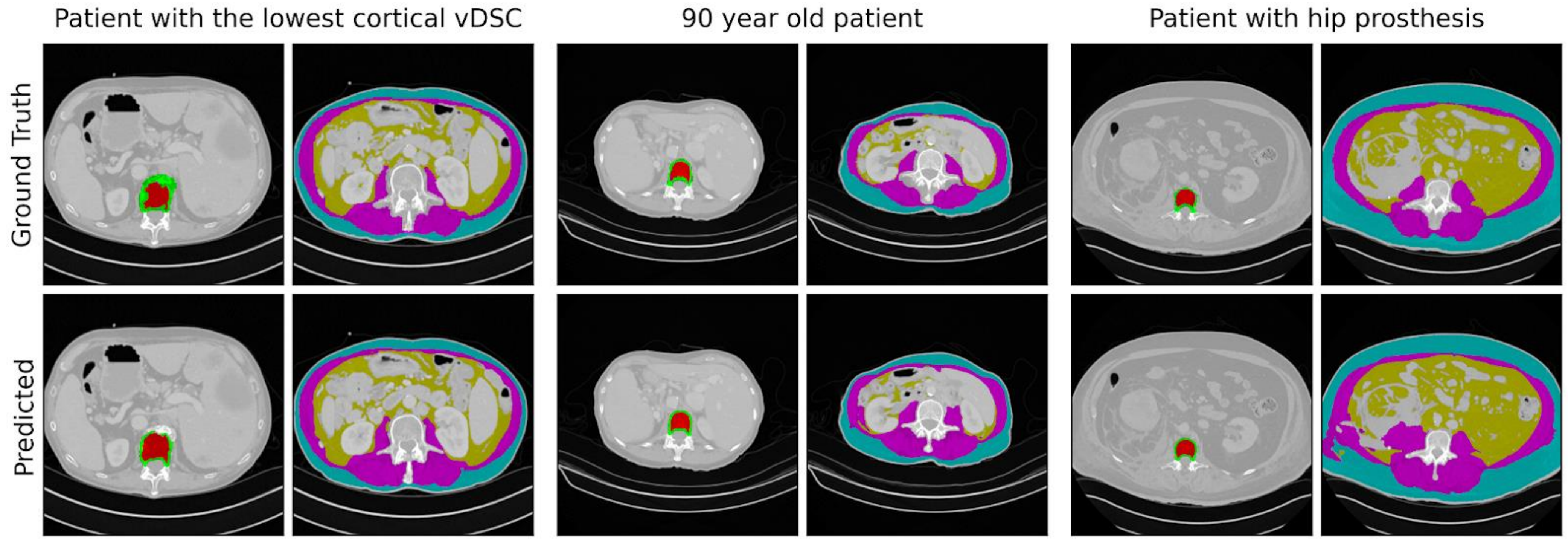

Fig. S6. Segmentations for three representative subjects from the independent test set. The left panel shows the subject with the lowest model performance for L1 cortical segmentation (vDSC=0.53 for the cortical region), the center panel depicts the oldest subject in the dataset, and the right panel highlights a subject with a hip prosthesis. The top row displays the ground truth segmentations, while the bottom row shows the segmentations predicted by the models.

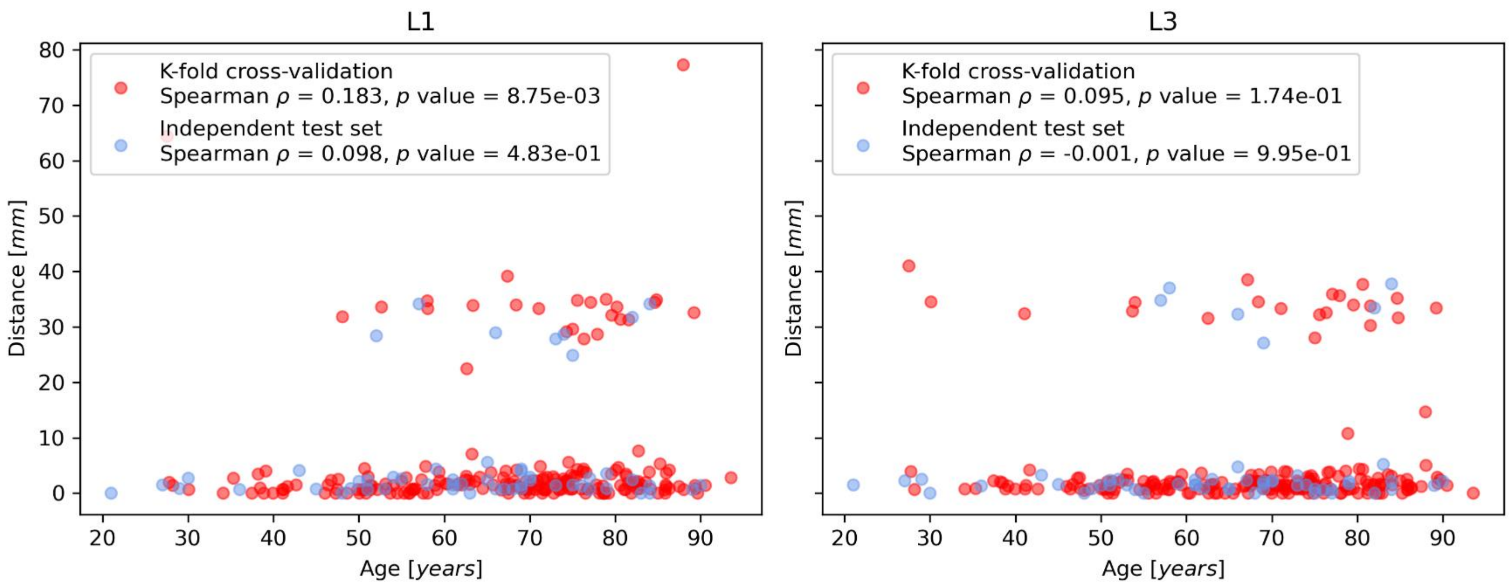

Fig. S7. Detection performance of L1 and L3 vertebrae centers, measured as the absolute distance (mm) between ground truth and predicted centers, as a function of patient age on the independent test set (blue) and on the k-fold cross-validation (red). Spearman's correlation coefficient (ρ) and associated *p* values are reported to assess the relationship between age and detection performance.

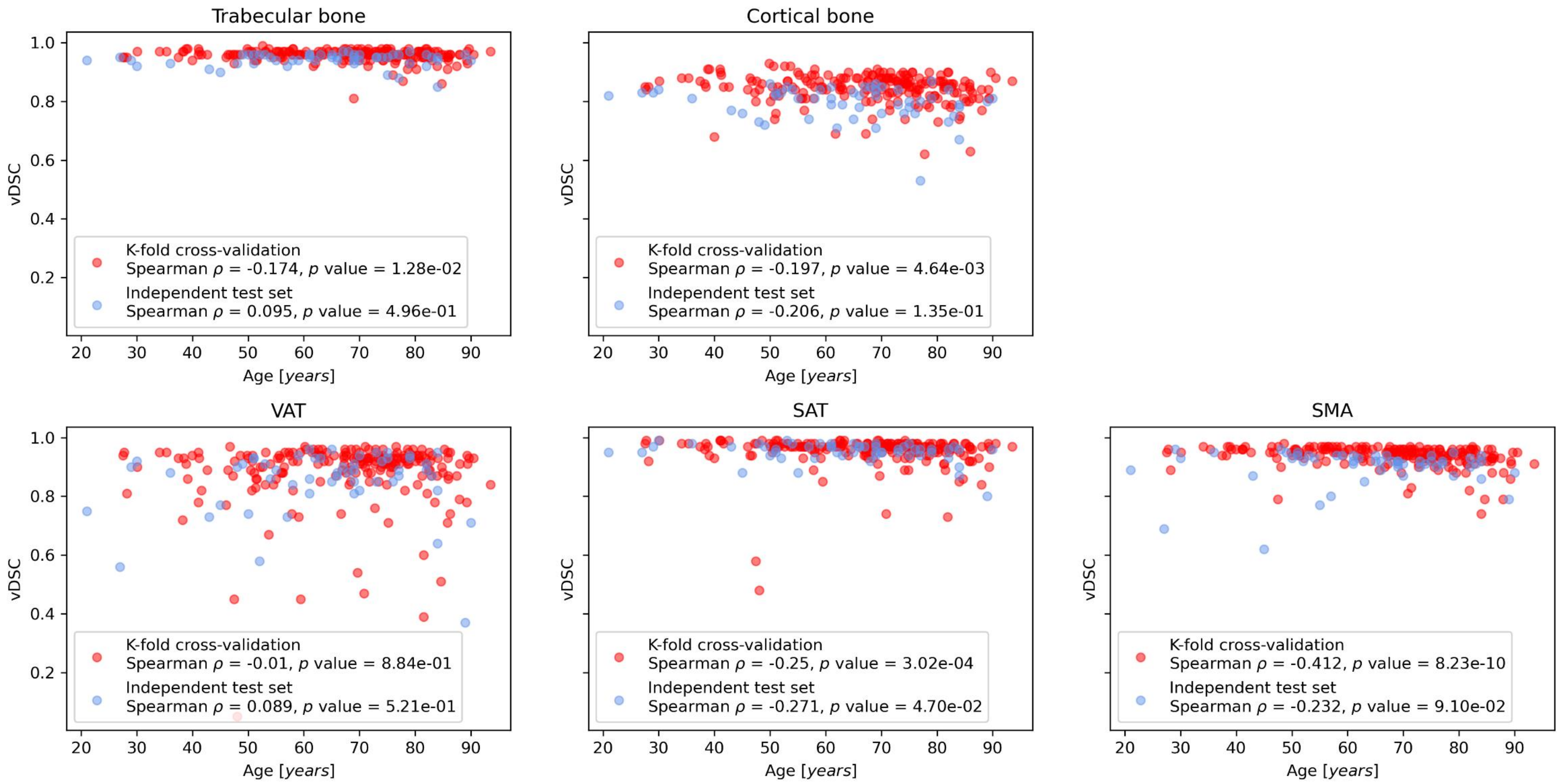

Fig. S8. Segmentation performance of U-net$_{L1}$ and U-net$_{L3}$ models, measured by vDSC, as a function of patient age on the independent test set (blue) and on the k-fold cross-validation (red). Spearman's correlation coefficient (ρ) and associated *p* values are reported to assess the relationship between age and segmentation accuracy.
